# Physiological and biochemical responses of soft coral *Sarcophyton trocheliophorum* to doxycycline hydrochloride exposure

**DOI:** 10.1038/s41598-023-44383-1

**Published:** 2023-10-17

**Authors:** Wenxin Xu, Waqas Ahmed, Mohsin Mahmood, Weidong Li, Sajid Mehmood

**Affiliations:** 1 College of Ecology and Environment, Hainan University, Haikou, 570228 China; 2 Key Laboratory of Agro-Forestry Environmental Processes and Ecological Regulation of Hainan Province, Hainan University, Haikou, 570228 China

**Keywords:** Ocean sciences, Marine biology

## Abstract

In light of the rapid expansion of the marine aquaculture industry, there has been widespread and irregular usage of aquatic drugs to combat biological diseases, which significantly impact the neighboring aquatic ecosystems. This study delves into the impact of the antibiotic aquatic drug known as doxycycline hydrochloride (DOX) on offshore soft corals, providing valuable data for the responsible use and management of aquatic drugs. In this investigation, we subjected *Sarcophyton trocheliophorum* to acute exposure to varying concentrations of DOX (0, 1, 5, and 10 mg L^−1^). We meticulously assessed critical parameters and observed alterations in protein levels, superoxide dismutase (SOD) activity, catalase (CAT) activity, lipid peroxidation (LPO), malondialdehyde (MDA) levels, Acid phosphatase (ACP) activity, alkaline phosphatase (AKP) activity, glutathione (GSH) concentration, glutathione S-transferase (GST) activity, glutathione Peroxidase (GSH-Px) activity, zooxanthellae density, and chlorophyll content. Our findings reveal that in the presence of DOX-induced environmental stress, there is a significant increase in LPO, MDA, chlorophyll, carotenoid levels, and the activities of ACP, GST, and GSH-Px in soft corals. Simultaneously, there is a noteworthy decrease in zooxanthellae density. Additionally, the protein concentration and SOD activity in soft corals experience substantial reduction when exposed to 5 mg L^−1^ DOX. Notably, CAT activity varies significantly in environments with 1 and 10 mg L^−1^ DOX. Moreover, these conditions exhibit a discernible influence on AKP activity, GSH content, and chlorophyll levels. These findings suggest that DOX exposure carries the potential for toxicity in aquaculture settings, affecting protein synthesis in soft corals and influencing oxidative stress, lipid peroxidation, immunity, and detoxification processes within these organisms. There is also a risk of compromising the coral defense system, potentially leading to coral bleaching. Furthermore, this study underscores the significant impact on photosynthesis, growth, and the metabolic dynamics of the coral-zooxanthellae symbiotic system. Consequently, our research offers vital insights into the mortality and bleaching effects of aquatic drugs on marine corals, offering a foundation for the prudent use and management of such substances.

## Introduction

With the significant growth of the aquaculture industry, global fisheries and aquaculture production have reached a record high. According to The State of World Fisheries and Aquaculture (SOFIA), the total output of fisheries and aquaculture reached 214 million tons in 2020. Asian countries accounted for 70% of the global total output of aquatic animals in the fisheries and aquaculture industry, and it is expected that the aquaculture industry will further expand in the next decade, with cultured fish taking up a greater share of consumption and trade^1^. In order to improve the breeding environment, eliminate non-breeding objects, prevent diseases and insect pests, and promote the growth or reproduction of cultured animals, many aquatic drugs are used in multiple processes such as seedling breeding, cultivation, and feeding^2^ . However, due to the large scale of aquaculture, drug abuse, and residues, large amounts of drugs are often consumed and a large amount of aquaculture wastewater containing high concentrations of antibacterial drugs is generated^3^. It is reported that the absorption rate of antibiotics in veterinary drugs is low, with up to 30% to 90% of antibiotics being excreted through the feces in their original form^4,5^. Due to incomplete absorption and metabolism, it is estimated that up to 54,000 tons of antibiotics are excreted by humans and animals, and ultimately 53,800 tons of antibiotics enter the aquatic environment after various wastewater treatments^6^.

Coral reefs are among the most bio-productive and diverse ecosystems on earth that serve as the foundation of marine fisheries and tourism in tropical and subtropical regions, playing an important role in social, economic, industrial and cultural aspects^7–9^. However, the combined effects of global warming, temperature reduction, freshwater inflow, and bacterial infections have caused a large amount of bleaching and death of coral reefs^10–12^, so that tropical shallow sea ecosystems such as coral reefs may face the same rapid decline fate as tropical terrestrial ecosystems such as tropical rainforests. Large-scale coral bleaching has increased year on year over the past few decades^13^ and it is expected that by 2040, there will be severe bleaching worldwide every year putting more than 90% of coral reefs at risk of long-term degradation^14,15^. It has been reported that antibiotic residues have been detected in the coastal and offshore coral reef waters of China^16–18^. Antibiotics can affect the sedimentation of coral larvae, making a negative impact on coral symbiotic relationships^19^, and coral symbiotic bacteria in the adjacent culture areas that are more resistant^20^.

Doxycycline hydrochloride (DOX) is one of the most commonly used antibiotics in food animals^21^. It has become one of the most important anti-infective drugs in aquaculture and is widely used in the prevention and treatment of human and animal diseases^22^. Statistics show that the total consumption of DOX is about 3810 tons in China^6^. In mariculture cage farms in Vietnam, an average of 78.8 g of DOX is used for disease control in lobster culture per ton^20^. More studies in China and other countries have reported the presence of DOX in rivers, lakes and seas, animal feces and soils^23,24^, which will lead to high ecological risks in the water environment^22^. In addition to direct contamination, potential environmental pollutants and their bioactive metabolites can continuously enter lakes and oceans through a variety of pathways^25^. It is reported that DOX is a highly toxic drug for sea bass juveniles and has obvious effects such as death and developmental deformities of zebra fish^26,27^, inhibitory effects on the growth of algae, photosynthesis and the synthesis of proteins and enzymes^28,29^. While DOX plays a significant role in medical care and feed additives, it may also be directly or indirectly contaminated into the aquatic environment, causing certain toxicity to aquatic organisms. Considering the long-term bioaccumulation and combined toxicity of antibiotic aquatic drugs to marine organisms, the ecological risk of aquatic drug pollution cannot be ignored^22^.

Healthy corals primarily fulfill their energy requirements by harnessing photosynthesis from symbiotic algae residing within their tissues. However, coral bleaching results in a substantial reduction in the products of algal photosynthesis and is accompanied by profound physiological transformations^30^. Soft corals contain photosymbionts that provide energy for the host, the zooxanthellae density and chlorophyll content are important indicators of photosynthesis and albinism in coral symbiotic systems^31,32^. Coral physiological index changes can be used as an effective indicator for coral bleaching studies^30^, protein content corresponds to the protein synthesis ability of coral, catalase (CAT) and superoxide dismutase (SOD) can represent coral oxidative stress levels^33^, lipid peroxidation (LPO) and malondialdehyde (MDA) are commonly used indicators to evaluate lipid peroxidation and oxygen free radical scavenging capacity^34^, acid phosphatase (ACP) and alkaline phosphatase (AKP) play important roles in biological immunity and metabolism processes^35^, reduced glutathione (GSH), glutathione S-transferase (GST) and glutathione Peroxidase (GSH-Px) has an important detoxifying function for corals^36^. To the best of our knowledge, there are few or no relevant research reports on the effects of the aquatic drug DOX on corals.

In this study, we focused on the examination of a prevalent soft coral species found in Sanya, Hainan province, namely "*Sarcophyton trocheliophorum*". The aim was to evaluate the impact of doxycycline hydrochloride (DOX) contamination on this coral species by meticulously measuring alterations in key physiological, biochemical, and symbiotic indicators. The research not only contributes valuable insights into the effects of aquatic drugs on coral health but also serves as a crucial data reference for guiding coral protection and restoration efforts in the region.

## Materials and methods

### Corals

**Figure 1:**
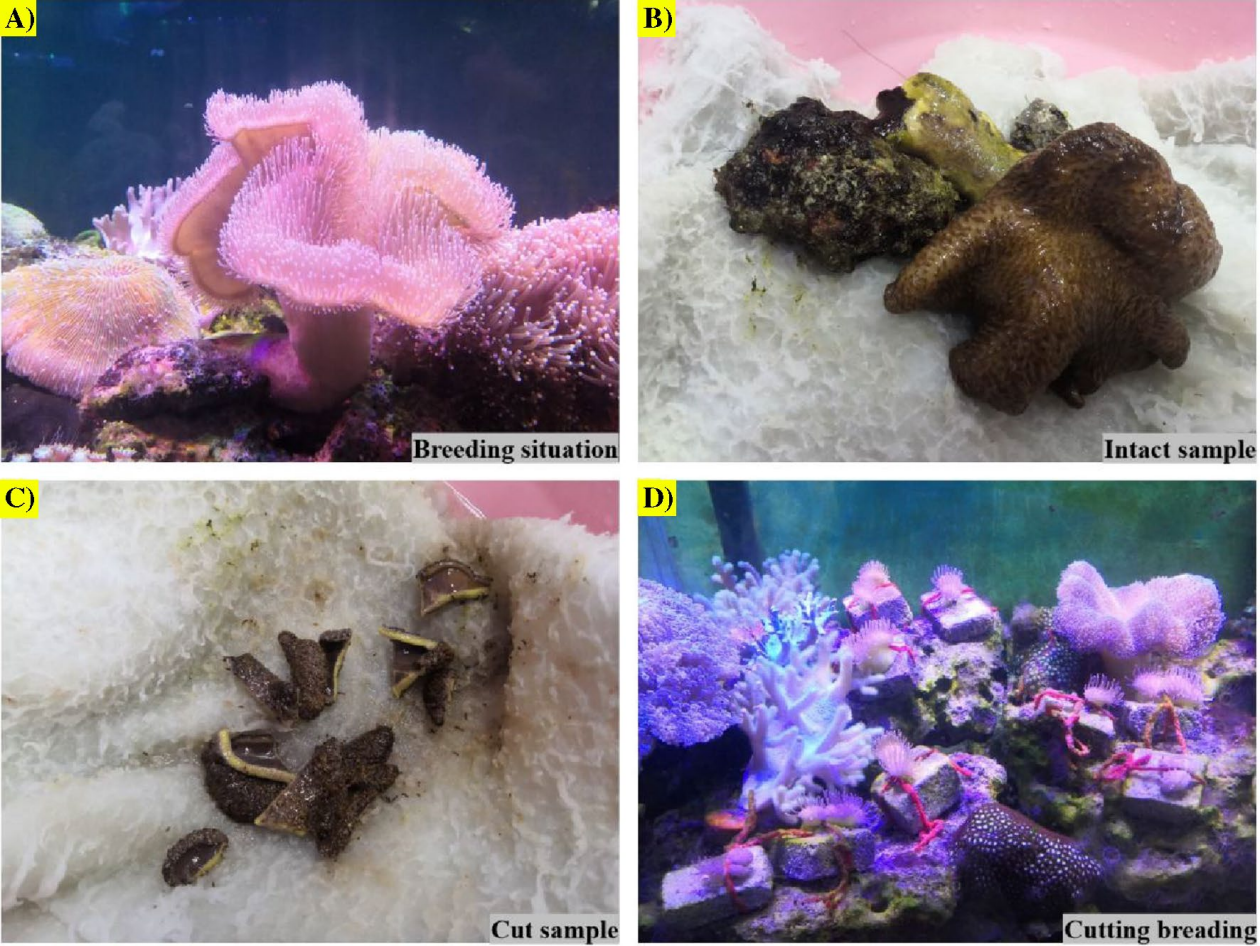
Coral sample and cutting breeding processes.

A soft coral was gathered from the southeast of Xidao waters (located at ~ 5 m depth) in Sanya, China, and transported back to the laboratory for acclimation, recovery and temporary breeding in a glass aquarium with a size of 70 cm × 60 cm × 50 cm (Fig. 1A–D). The temporary breeding conditions were a water temperature of 25 ± 3 °C, a salinity of 30‰, a light–dark ratio of 12:12, and a water flow cycle of 2000 L/h, the water flow was provided by the circulating pump and the wave making pump were installed in the aquarium^37,38^. After the full extension of coral tentacles, a 5 cm-sized tissue was separated and adhered to as stone and was placed back in the aquarium were acclimated for 4 weeks.

### Experimental design

Doxycycline hydrochloride (≥ 98%) was purchased from Shanghai Macklin Biochemical Corporation Limited (Shanghai, China). In order to set the DOX stress concentrations, the soft corals were exposed to the different concentrations of DOX environments refer to the methods of Wu^39^, according to the status of tentacle extension, color change and mucus production of soft coral determined the DOX concentrations of experimental treatments. Refer to the pre-experimental results, three different concentrations of DOX were selected as control (0 mg L^−1^), low (1 mg L^−1^), medium (5 mg L^−1^), and high (10 mg L^−1^). Three randomly selected pieces of coral with normal tentacle vitality were placed in each aquarium, except for different treatment conditions, other breeding conditions were consistent with the temporary rearing method and a wave making pump was added to each individual aquarium, and the 8-h acute toxicity experiment under environmental stress of DOX was initiated after the temporary rearing coral tentacle stretch was normal and the state was stable. After this experiment, the samples were rinsed with 1× PBS (0.01 M) and stored in sterilized centrifuge tubes at − 80 ℃ for subsequent detection.

### Protein and oxidation resistance enzyme assays

According to the manufacturer's recommendations, weigh the weight of coral tissue, add 10 times of pre-cooled physiological saline in the ratio of weight (g) to volume (ml) = 1:10, homogenize with a high-speed grinder at 2500 rpm, centrifuge for 10 min, take the supernatant, use the total protein quantitative assay kit (A045-2, Nanjing Jiancheng, China), dilute with two times of physiological saline, and measure the OD value at a wavelength of 595 nm. The total Superoxide Dismutase (T-SOD) assay kit (A001-1, Nanjing Jiancheng, China) was used to determine the OD value at a wavelength of 559 nm using a fivefold dilution of physiological saline. The catalase (CAT) assay kit (A007-1, Nanjing Jiancheng, China) was used to determine the OD value at a wavelength of 405 nm, and the protein concentration, SOD, and CAT activity of coral tissue samples were calculated.

### Lipid peroxidation and immunocompetence indicators measurement

According to the manufacturer's instructions, we used the lipid Peroxide (LPO), malondialdehyde (MDA), and acid phosphatase (ACP) assay kit (KT50571-B, KT7878-B, KT4958-B, Jiangsu Kete, China) to determine the OD value at 450 nm, respectively, and used the alkaline phosphatase (AKP) assay kit (A059-2, Nanjing Jiancheng, China) to determine the OD value at 520 nm, and calculate the LPO, MDA content, and ACP, AKP activity of coral tissue samples.

### Detoxification ability estimation

Following the kit's instructions, 0.1 ml of tissue homogenate supernatant was extracted. A reduced glutathione (GSH) assay kit (deproteinization method) labeled as A006-2, originating from Nanjing Jiancheng, China, was utilized. To this, 0.1 ml of reagent was added and thoroughly mixed. The mixture was then centrifuged at 3500 rpm for 10 min. Subsequently, the supernatant was collected and its optical density (OD) was measured at 405 nm. Furthermore, the levels of glutathione S-transferase (GST) and glutathione peroxidase (GSH-Px) were determined using kits labeled as MM-36002O2 and KT50020-B, respectively, both provided by Jiangsu Kete, China. The OD value was measured at a wavelength of 450 nm to calculate the GSH content and assess the activities of GST and GSH-Px in the coral tissue samples.

### Symbiotic relationship and photosynthetic efficiency determination

Refer to the methods of Chen^37^ and optimize it, the variation of zooxanthellae density in soft corals after DOX exposure was studied. Weigh the wet weight of coral tissue and transfer it to a hand-held homogenizer to add nine times the volume of 1× PBS (0.01 M) buffer is made into 10% tissue homogenate, filter with a 40 μm cell sieve to obtain the filtrate of the insect yellow algae, and take 10 μL of filtrate is counted in a blood cell counting plate, the density of symbiotic zooxanthellae is calculated based on the number of zooxanthellae per milliliter of tissue homogenate (cells/mL). Chlorophyll (*a* + *b*), chlorophyll *c*, and carotenoid content were measured using the chlorophyll assay kit (A147-1, Nanjing Jiancheng, China), the chlorophyll c assay kit (KT50568-B, Jiangsu Kete, China), and the carotenoid assay kit (KT50013-B, Jiangsu Kete, China) according to the specification of these kits.

### Statistical analysis

The experimental data were organized, mapped, and statistically analyzed using GraphPad Prism 9. One-way analysis of variance (ANOVA) followed by multiple comparison tests with the LSD test was performed to determine the significance of the different results. The descriptive statistics are expressed as mean ± SEM, experimentation was carried out in triplicate with *p* < 0.05 as a significant difference.

## Results

### Protein concentration and SOD, CAT activity

**Figure 2:**
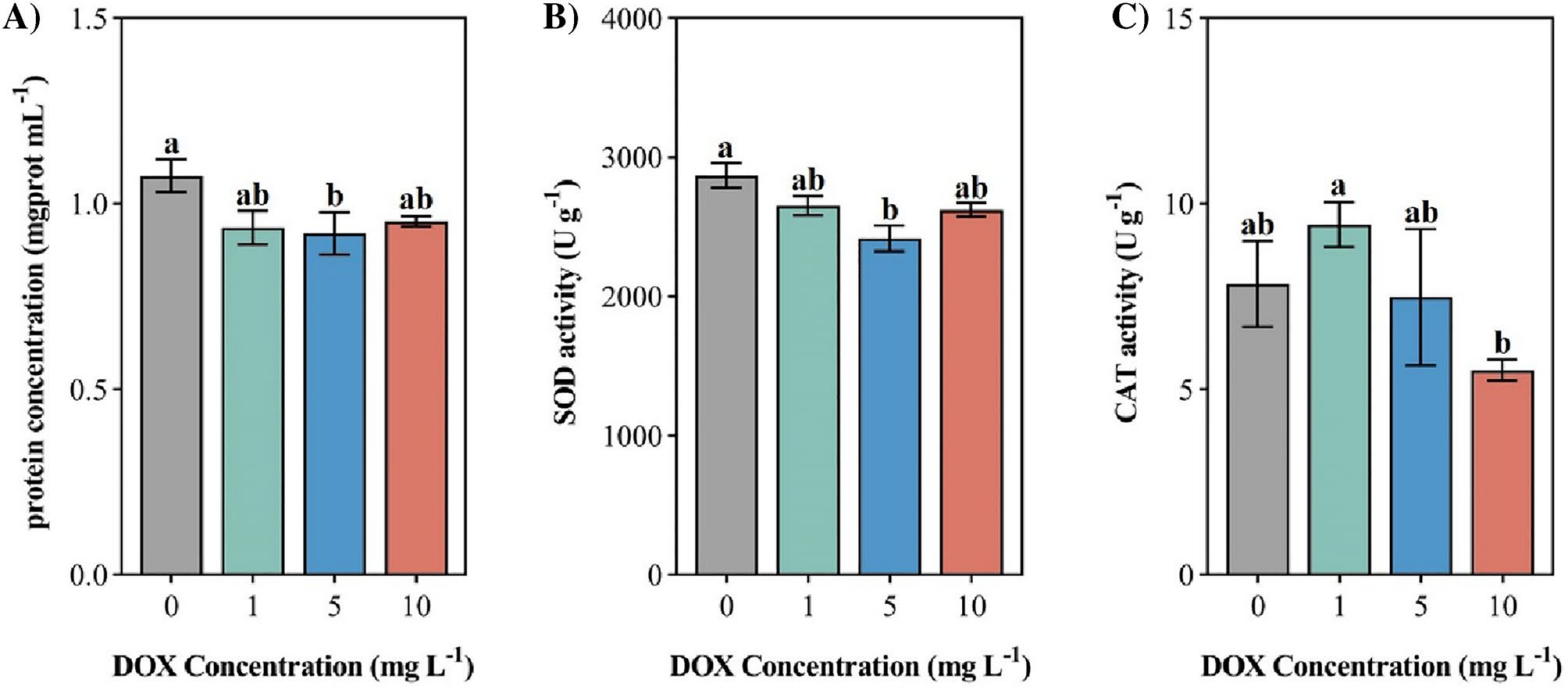
Effects of different levels of DOX of corals on (**A**) protein concentration; (**B**) SOD activity; (**C**) CAT activity. Different letters indicate significant differences (*P* < 0.05, n = 3).

**Figure 3:**
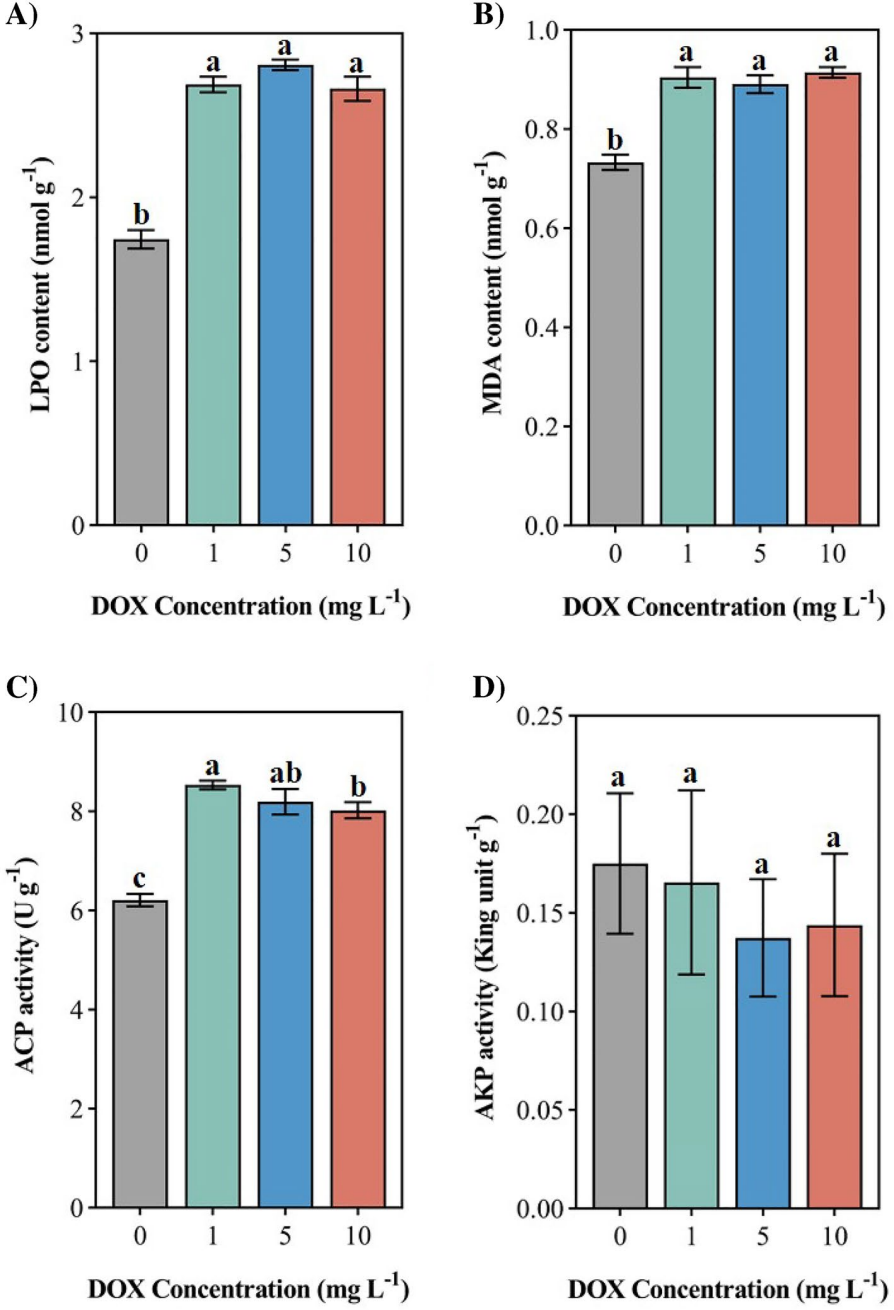
Effects of different levels of DOX of corals on (**A**) LPO content; (**B**) MDA content; (**C**) ACP activity; (**D**) AKP activity. Different letters indicate significant differences (*P* < 0.05, n = 3).

The protein concentration of soft coral was significantly reduced [(0.92 ± 0.06) mg. prot/mL] (Fig. 2A, P < 0.05) under the medium level of DOX (5 mg L^−1^) and was reduced compared with the control level by 14.38%, then the low (1 mg L^−1^) and high (10 mg L^−1^) levels of DOX environments did not have a significant effect on the soft coral protein concentrations but were reduced compared with the control level by 12.96% and 11.33%, respectively. After exposure to the medium level of DOX (5 mg L^−1^) environment, the SOD activity of soft corals was significantly decreased [(2417 ± 93.81) U g^−1^] (Fig. 3D, P < 0.05), which was reduced by 15.74% compared with the control level. The SOD activity of soft corals exposed to the low (1 mg L^−1^) and the high (10 mg L^−1^) levels of the DOX environment also decreased compared with the control group. After exposure to the DOX environment, the CAT activity of soft corals differed significantly in multiple comparisons of the low (1 mg L^−1^) and the high level (10 mg L^−1^) of DOX environments (Fig. 3C, P  < 0.05). Among them, soft corals exposed to the low level (1 mg L^−1^) of DOX environment had the highest CAT activity [(9.44 ± 0.61) U g^−1^], and soft corals exposed to the high level of DOX (10 mg L^−1^) had the lowest CAT activity [(5.53 ± 0.29) U g^−1^]. Under the stress of DOX at different concentrations, the CAT activity of soft corals was promoted first and then inhibited, among which the CAT activity of soft corals exposed to the low level of DOX (1 mg L^−1^) increased by 20.37% compared with the control level, which showed a promoting effect, and the CAT activity of soft corals exposed to the medium (5 mg L^−1^) and the high (10 mg L^−1^) levels of DOX environments was reduced by 4.51% and 29.55% respectively compared with the control level, showed an inhibitory effect.

### LPO, MDA content and ACP, AKP activity

After exposure to the DOX environment, the LPO content was significantly increased in all levels of DOX environments and compared with the control level by 54.04%, 60.92% and 52.58%, respectively (Fig. 2B , P < 0.05). There was a significant increase in the MDA content in soft corals after exposure to different levels of DOX environments and had the highest MDA content [(0.91 ± 0.01) nmol g^−1^] in high (10 mg L^−1^) level of DOX environment (Fig. 2C , P  < 0.05), among which the MDA content exposed to the low (1 mg L^−1^), medium (5 mg L^−1^) and high (10 mg L^−1^) levels of DOX environment increased by 23.33%, 21.52% and 24.70% respectively compared with the control level. The ACP activity of soft coral exposed to all levels of DOX was significantly increased (Fig. 3A , P < 0.05), and the ACP activity of soft coral exposed to the low level of DOX (1 mg L^−1^) environment was the highest [8.53 ± 0.09 U g^−1^]. Under different concentrations of DOX stress, the ACP activity was significantly increased compared with the control level by 37.31%, 31.83% and 29.11%, respectively. Under the stress of different levels of DOX, the AKP activity of the soft coral was inhibited but the difference was not significant (Fig. 3B). Among them, the AKP activity of the soft coral exposed to the medium level of DOX (5 mg L^−1^) was the lowest and 21.53% lower than the control level, as well as the AKP activity of the soft coral exposed to the high level of DOX (10 mg L^−1^) was reduced compared with the control level by 17.78%.

### GSH content and GST, GSH-Px activity

**Figure 4:**
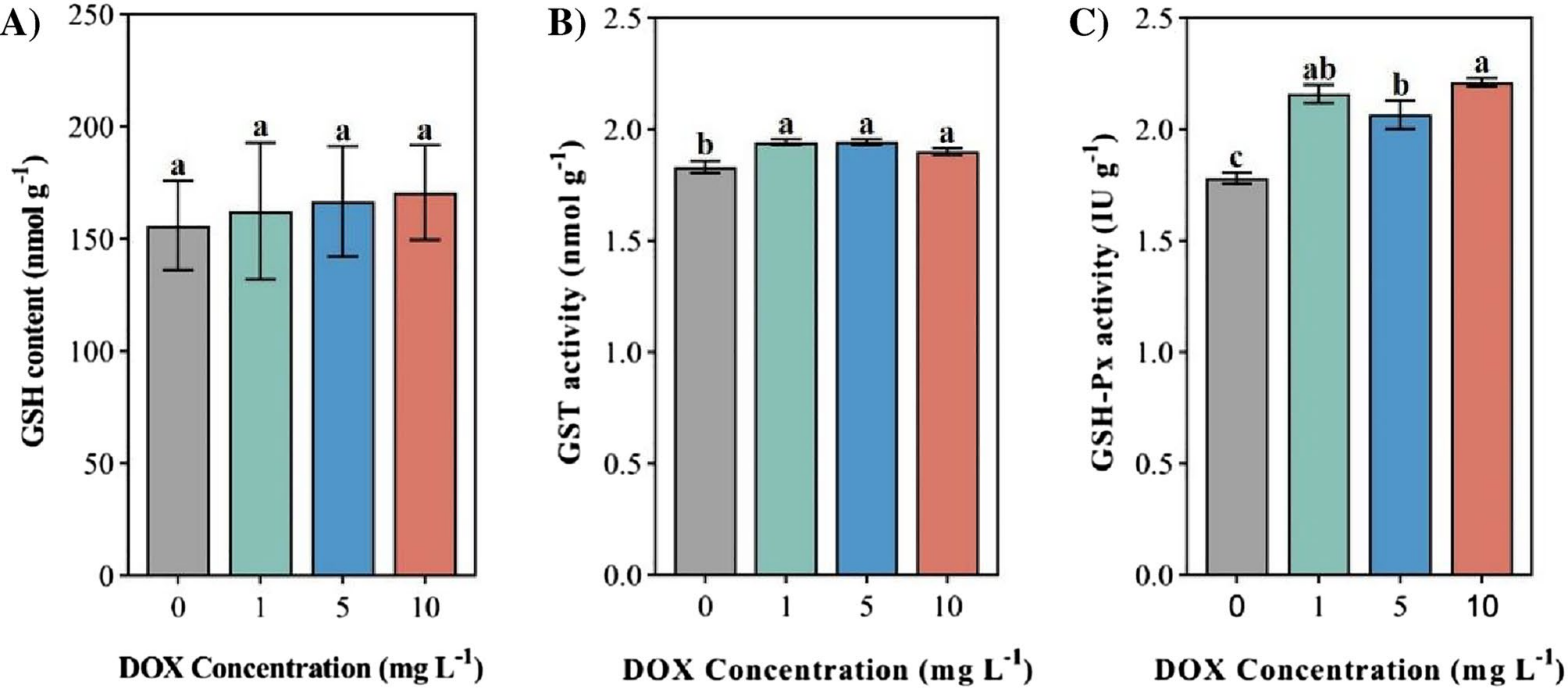
Effects of different levels of DOX of corals on (**A**) GSH content; (**B**) GST activity; (**C**) GSH-Px activity. Different letters indicate significant differences (*P* < 0.05, n = 3).

After exposure to DOX, there was no significant effect despite an increase in GSH content in individual individuals of soft corals (215.60 nmol g^−1^) (Fig. 4A). The GSH content of soft corals exposed to the high level of DOX (10 mg L^−1^) was the highest, higher than that of the control group by 9.39% and the GSH content of soft corals showed a stepwise promoting effect in different DOX treatment groups. Under the stress of DOX, the GST activity of soft coral increased significantly (Fig. 4B, P  < 0.05), and the GST activity of soft coral showed significant promotion in different DOX treatment levels. The GSH-Px activity of soft corals exposed to the DOX environment increased significantly (Fig. 4C, P < 0.05). The GSH-Px activity of soft corals exposed to the high level of DOX (10 mg L^−1^) was the highest [2.21 ± 0.02 IU g^−1^], which was significantly higher than that of the control level by 24.13%.

### Zooxanthellae density and chlorophyll content

**Figure 5:**
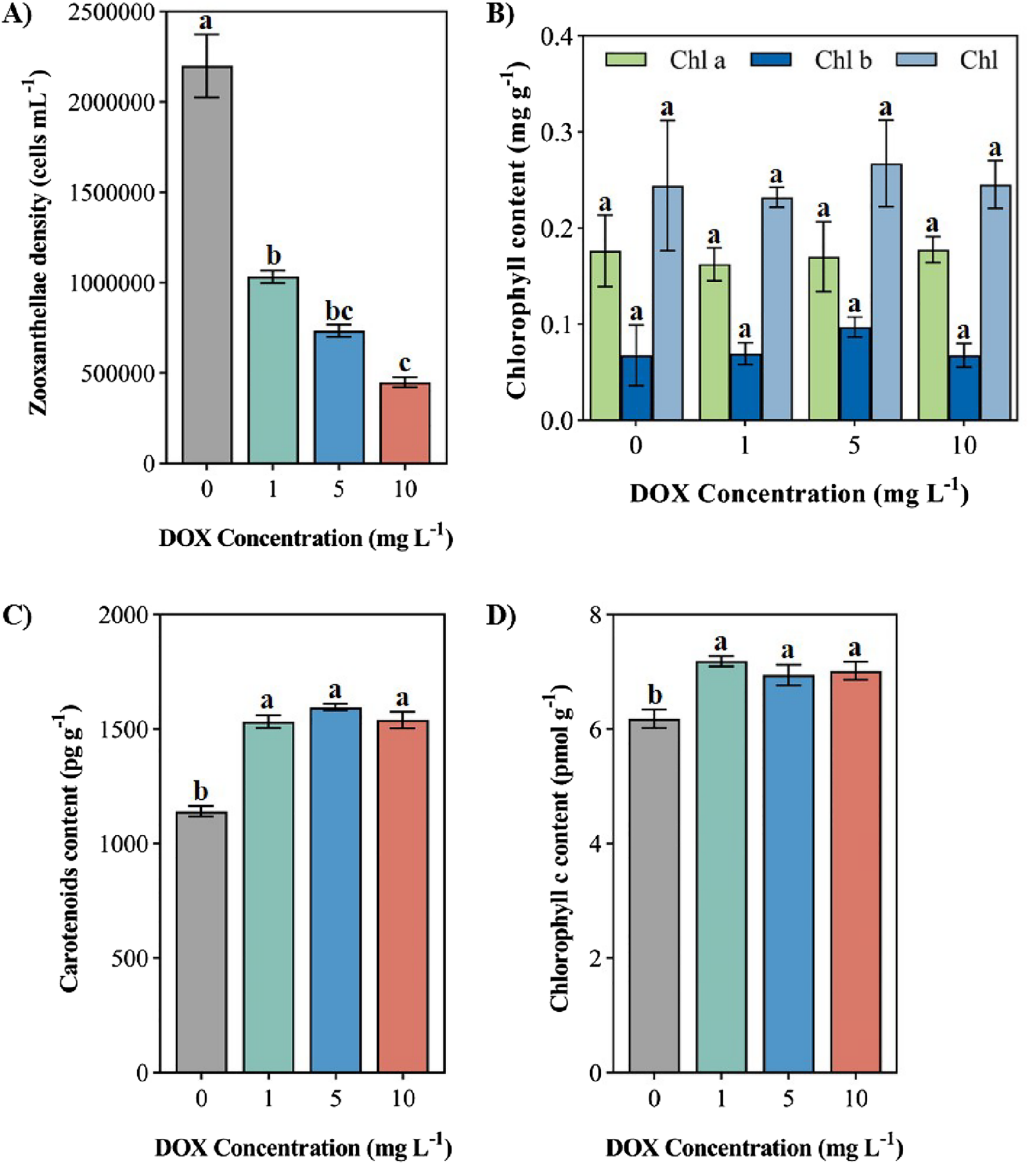
Effects of different levels of DOX of corals on (**A**) zooxanthellae density; (**B**) Chlorophyll (*a* + *b*) content; (**C**) carotenoids content; (**D**) Chlorophyll *c* activity. Different letters indicate significant differences (*P* < 0.05, n = 3).

There was a significant effect in the zooxanthellae density in soft coral under all levels of DOX environments (Fig. 5A, P < 0.05), among which the zooxanthellae density of soft coral exposed to the high level of DOX (10 mg L^−1^) is the lowest [450000 ± 28,868 cells mL^−1^]. Compared with the control level, the zooxanthellae density in soft coral under different levels of DOX environments decreased by 53.03%, 66.67% and 79.55% respectively and showed a significant gradient inhibition effect. DOX did not have a significant effect on chlorophyll content in soft corals (Fig. 5B). Different concentrations of DOX on the chlorophyll content of soft corals showed the effect of first inhibition and then promotion, of which the chlorophyll content of soft corals exposed to the low level of DOX (1 mg L^−1^) was the lowest, which was 7.92% lower than that of the control level. Under the pressure of DOX, the chlorophyll *b* content of soft corals was increased and showed a promoting effect, of which Chlorophyll *b* content of soft corals exposed to the medium level of DOX (5 mg L^−1^) was the highest in the environment of DOX, which was 43.29% higher than that of the control level. The chlorophyll content of soft corals showed a trend of first decreasing and then increasing similar to the content of chlorophyll *a*, meanwhile which the chlorophyll content of soft corals exposed to the low level of DOX (1 mg L^−1^) was the lowest, which was 5.00% lower than that of the control group, and the chlorophyll content of soft corals exposed to the medium level of DOX (5 mg L^−1^) environment was the highest, 9.41% higher than that of the control level. The carotenoids content of soft coral exposed to different levels of DOX increased significantly (Fig. 5C , P < 0.05), among which the carotenoids content of soft corals exposed to the medium level of DOX (5 mg L^−1^) was the highest [1596 ± 14.61 pg g^−1^], which was 39.88% higher than the control level, and the carotenoids content of soft coral exposed to the low (1 mg L^−1^) and high (10 mg L^−1^) levels of DOX environments was increased by 34.33% and 34.98% respectively compared with the control level. After exposure to the DOX environment, the chlorophyll c content in soft corals increased significantly (Fig. 5D, P  < 0.05). The chlorophyll c content in soft corals exposed to the low (1 mg L^−1^), medium (5 mg L^−1^) and high (10 mg L^−1^) levels of DOX environments was significantly increased by 16.23%, 12.31% and 13.51% respectively compared with the control level and showed a significant promoting effect.

## Discussion

With the development of fisheries and aquaculture, a large number of drugs enter the marine environment through direct or indirect means^25^, furthermore has an impact on marine life. Prata^29^ reported the toxicity of DOX inhibits the synthesis of *Tetraselmis chuii* protein, and the results of this experiment show that all levels of DOX environments can reduce the protein concentration of soft corals, and it is speculated that DOX may inhibit the synthesis of soft coral protein. Under stress conditions, the production of reactive oxygen species (ROS) in corals is thought to be the mechanism that leads to cell damage and the disappearance of symbiotic populations^40^. SOD constitutes the first line of defense against ROS in the antioxidant system^41^, it converts reactive oxygen radicals from the polyp into hydrogen peroxide, which is further scavenged by CAT^42,43^, Wu^39^ reported that Changes in SOD and CAT activity of *Microcystis aeruginosa* under DOX stress. Similar to its findings, SOD and CAT activity was also stimulated in this study, indicating that coral cells were under oxidative stress. SOD inhibition and CAT facilitation were observed in the low level of DOX environment (1 mg L^−1^), which may be caused by the damage of antioxidant system and the stimulation of high concentration of hydrogen peroxide in coral cells. It is reported that the decrease of CAT may be accompanied by the parallel decrease of SOD, because the two enzymes are functionally linked and tandem^44^, similar to the report, in this study, the decreased activities of SOD and CAT were observed in the medium (5 mg L^−1^) and high (10 mg L^−1^) levels of DOX environments suggested that DOX stress might reduce the metabolic activity of soft corals. This is contrary to the report from Krueger^40^ that high-temperature stress promotes the metabolic activity of scleractinian corals, it is speculated that the corresponding stress mechanism and metabolic function of coral may have conditions and interspecific specificity, soft corals may have bleaching susceptibility to DOX contamination. DOX causes some degree of oxidative damage to soft corals.

LPO is an important indicator reflecting the imbalance between reactive oxygen species ROS and antioxidants, the increase of LPO content in the short term indicates that ROS initiates the lipid peroxidation process in coral, thus weakening its antioxidant defense ability^45^. In this experiment, the LPO content of soft coral exposed to the DOX environment increased significantly, indicating that its antioxidant defense ability was damaged. The significant increase in LPO content in soft coral may be related to the decrease of SOD and CAT activity or the increase in GSH content^46^. It is speculated that the physiological changes caused by oxidative stress may have adverse effects on its development and growth. DOX can induce coral lipid peroxidation in a short period of time, which has an obvious negative impact on the antioxidant defense ability of coral. MDA is one of the final products of lipid peroxidation in the cell membrane and an important indicator of the degree of coral lipid peroxidation and cellular oxygen-free radical damage^47^. Increased MDA implies endogenous oxidative damage and lipid peroxidation^48^, Wu ^39^ reported that DOX stress could improve the MDA content of *M. aeruginosa*, similar to the existing research results, the MDA content of soft coral in this study increased significantly, indicating that DOX can cause lipid peroxidation of soft coral and seriously damage cells. Under stress conditions, the production of MDA is usually accompanied by the consumption of antioxidant metabolic enzymes such as SOD and CAT, while further inducing the production of oxygen free radicals, inhibiting the antioxidant system, and aggravating the damage of coral cells^47^. In this experiment, the MDA content in the high level of DOX (10 mg L^−1^) is the highest, and the high levels of DOX environment led to the decline of SOD and CAT activity, which may be the main reason for DOX to cause coral cell damage. Acute exposure to DOX can cause coral lipid peroxidation, inhibit the antioxidant system, and cause severe cell damage. AKP and ACP play a key role in the decomposition and assimilation of nutrients, the digestion and detoxification process and the self-digestion of dead cells^49^. AKP is an important reference indicator of coral immune function and health status^50^. Studies have shown that AKP activity was determined by its optimal pH value, and its activity decreased when the tissue was seriously damaged^45^, similar to previous studies, the AKP activity of soft corals in this study decreased, which indicated that DOX stress changed the pH in soft corals, which may cause serious damage to the structure of its tissues and organs. ACP is a typical lysosomal enzyme, which plays a role in killing and digesting microbial pathogens in the process of the immune response^51^, the results of this experiment showed that the ACP activity of soft coral increased significantly, indicating that acute DOX exposure enhanced the digestion and detoxification process of soft coral^48^, which may be a stress response measure for coral to respond to acute stress environments. DOX can trigger an immune response in corals in the short term, impact their immune capacity and health status, and even cause serious damage to tissues and organs.

GSH is the most important non-enzymatic antioxidant in the organism^52^, and has important detoxification and protection effects with GST^53^. The levels of DOX were positively correlated with the GSH content in soft coral, which may be an antioxidant mechanism to protect coral from oxidative stress or detoxification. This conclusion has been confirmed in relevant oxidative stress experiments^46^. GST is the key phase II metabolic enzyme in the detoxification process of all eukaryotes^54^, which can protect tissues from oxidative stress by promoting the conjugated reaction of exogenous deleterious substances with GSH^55^. In this study, GST activity increased significantly, indicating that DOX can trigger the detoxification system of coral in a short time, and acute DOX exposure can promote the detoxification function of soft coral, which is consistent with the results of Jiang^45^. GSH-Px can inhibit cell apoptosis by catalyzing the reduction reaction of GSH with hydrogen peroxide^56^. The results of this experiment show that the GSH-Px activity is significantly increased like the GST activity, indicating that the formation of GSH-related antioxidants may not be interfered by or have antagonistic effects on DOX^57^. In conclusion, soft coral can resist oxidative stress by regulating GSH, GST and GSH-Px levels.

Changes in zooxanthellae density and chlorophyll content of soft coral are important indicators of the response of coral symbiotic systems to environmental stress. After being stressed, the symbiosis system will actively expel zooxanthellae and cause coral bleaching^58^, The experimental results showed that the density of symbiotic zooxanthellae in soft coral exposed to DOX decreased significantly, and the zooxanthellae density decreased with the increase of DOX level, which indicated that DOX exposure might damage the symbiotic relationship between coral and zooxanthellae. The research shows that the reduction of the zooxanthellae density can offset the respiratory loss caused by partial stress conditions, and increase the light intensity of zooxanthellae in the deep layer of coral tissue to improve the photosynthetic efficiency, which is conducive to the survival of coral in the polluted environment^59^. It is speculated that the escape of zooxanthellae can be the self-protection mechanism of coral to cope with the harsh environment. Wu^39^ has confirmed that DOX has an inhibitory effect on the growth of *Microcystis aeruginosa*, Prata^28^ reported DOX exposure can increase the chlorophyll content of *Tetradermis chuii* in a short time, similar to existing research results^60^, the low (1 mg L^−1^) and medium (5 mg L^−1^) levels of DOX environments reduced the chlorophyll content of soft corals, indicating that DOX stress may lead to damage to the chlorophyll molecules of Coral symbiotic zooxanthellae, affecting the metabolism and photosynthesis process of algal cells, and thus endangering the growth and development process of soft corals. Under DOX stress, the chlorophyll b content of soft corals was increased, which may be a reaction of symbiotic algae to reduced light utilization, manifested by the shading effect common in symbiotes, which is consistent with the results from Falkowski^61^. Under the stress of the chlorophyll content of soft corals decreased, indicating that the low level of DOX (1 mg L^−1^) can reduce the chlorophyll content of zooxanthellae in soft corals so that its photosynthetic rate decreases and the medium (5 mg L^−1^) and the high (10 mg L^−1^) levels of DOX may have a promoting effect on its photosynthesis. In this experimental result, the chlorophyll *a* and chlorophyll content of soft corals showed a trend of first decreasing and then increasing, which is speculated to be a mechanism for soft corals to resist environmental stress in the short term, and DOX will have an effect on the photosynthesis of its symbiotic algae, the results are consistent with that from Schoepf^30^. The increase in chlorophyll content is a common response of algae to the reduction of light utilization efficiency^61^. In this study, chlorophyll c and carotenoid content in soft coral increased significantly, which may be the response of symbiotic algae to the reduction of light utilization efficiency. The decrease in light utilization efficiency and the increase in energy consumption force soft corals to supplement their energy supply by increasing the content of chlorophyll *c* and carotenoids, this serves as a temporary remedy for improving photosynthetic efficiency and adapting to the DOX environment. The chlorophyll *a* and chlorophyll *b* contents did not change significantly, while the chlorophyll *c* and carotenoids contents increased significantly, which may be due to the contents of chlorophyll *c* and carotenoids are more sensitive to DOX than the chlorophyll *a* and chlorophyll *b*. In summary, it is shown that acute DOX exposure disrupts the symbiotic relationship between zooxanthellae and corals in the short term, and has a significant impact on its photosynthesis and growth metabolism.

## Conclusion

In this study, a common soft coral species *S. Trocheliophorum* was selected and the effects of DOX were assessed. It is of great significance for the protection of marine aquatic life and biodiversity and puts forward constructive opinions and suggestions on the use and management of aquatic drugs in human production activities. This study revealed the following three points: first, DOX exposure has a potential toxic effect on aquaculture, which has an inhibitory effect on the protein synthesis of soft corals; second, soft coral may have bleaching sensitivity to DOX pollution, DOX stress has a significant impact on the oxidative stress, lipid peroxidation, immunity, and detoxification function of soft corals, which can easily inhibit its defense system and lead to serious consequences such as tissue, organ, and cell damage, endangering the health of coral; third, soft coral can resist oxidative stress caused by DOX exposure by regulating GSH related antioxidant levels in the short term; fourth, acute DOX exposure will disturb the coral-zooxanthellae symbiosis system, and have a significant impact on its photosynthesis and growth metabolism, and the significant escape of zooxanthellae from soft corals, the shading effect caused by the increase of chlorophyll *b* content, the trend of chlorophyll *a* and chlorophyll content decreasing first and then increasing and the significant increase in chlorophyll *c* and carotenoid content may be the autonomous regulation mechanism of corals resisting environmental stress in the short term. These findings employes us to play a major role in the prevention and control of diseases in the aquaculture industry while focusing on problems such as irregular use and drug residues, and at the same time, we can effectively promote the governance of aquatic drugs and the protection of marine organisms through the following measures: first, the popularization of rational use of drugs while aquatic drugs are widely used; The second is to focus on the negative impact on marine life and ecology after drug use, and follow up the improvement and implementation of related treatment systems such as sewage, environmental pollutants, and their biologically active metabolites. In the future, it is urgent to ensure that the analysis results are more objective and reasonable by increasing the types of drugs and corals and extending the experimental processing time. Meanwhile, the mechanisms of stress resistance of soft corals exposed to aquatic drugs can be studied at the transcriptional and protein levels.

## Data Availability

All data generated or analysed during this study are included in this published article.
